# Development and Validation of Two Optimized Multiplexed Serologic Assays for the 9-Valent Human Papillomavirus Vaccine Types

**DOI:** 10.1128/msphere.00962-21

**Published:** 2023-03-16

**Authors:** Katrina M. Nolan, Brent Seaton, Joseph Antonello, Yuhua Zhang, Lauren Cook, Krystyn Delfino, Leonard J. Rubinstein, Kyeongmi Cheon, Thomas Group, Alain Luxembourg, Sheri Dubey

**Affiliations:** a Merck & Co., Inc., Rahway, New Jersey, USA; b Q^2^ Solutions Vaccines, San Juan Capistrano, California, USA; University of Maryland School of Medicine

**Keywords:** 9-valent human papillomavirus vaccine, competitive Luminex immunoassay, human papillomavirus, immunogenicity, total immunoglobulin G immunoassay

## Abstract

Two multiplex immunoassays are routinely used to assess antibody responses in clinical trials of the 9-valent human papillomavirus (9vHPV) vaccine. The HPV6/11/16/18/31/33/45/52/58 competitive Luminex immunoassay (HPV-9 cLIA) and HPV6/11/16/18/31/33/45/52/58 total immunoglobulin G Luminex immunoassay are used for measurements of immunogenicity. Following their initial validation in 2010, both assays were redeveloped, and several parameters were optimized, including the coating concentration of virus-like particles, type of Luminex microspheres, serum sample and reference standard diluent, reference standard starting dilution and titration series, and vendor and concentration of the phycoerythrin-labeled antibodies. Validation studies evaluated the assay performance parameters, including the intra-assay precision (repeatability), intermediate precision, linearity, relative accuracy, and limits of quantitation. In addition, since maintaining a link to the original assays that were used in trials supporting vaccine licensure is critical, the assays were formally bridged to the previous assay versions by using individual patient sera from a 9vHPV vaccine clinical trial (*n* = 150 day 1 [prevaccination] samples; *n* = 100 month 7 [1 month post-last vaccine dose] and *n* = 100 month 36 [30 months post-last vaccine dose; antibody persistence] samples). The results of the validation studies indicate that both optimized assays are accurate, specific, and precise over their respective quantifiable ranges. There was a strong linear association between the new and previous versions of both assays. Assay serostatus cutoffs for the redeveloped assays were established based on the bridging studies and, for the HPV-9 cLIA, further refined, based on additional data from HPV vaccine clinical studies so as to align the seropositivity rates between assay versions.

**IMPORTANCE** Assay modernization is a key aspect of vaccine life cycle management. Thus, new, reoptimized versions of two 9vHPV immunoassays have been developed and validated for use in ongoing and future HPV vaccine clinical trials. These assays are suitable for use in high-throughput testing for HPV antibodies in serum samples. Bridging to previous versions of the assays allows for the continuous monitoring of immune responses across assay versions, including in immunogenicity studies that involve new populations as well as long-term follow-up studies.

## INTRODUCTION

Human papillomavirus (HPV) is responsible for approximately 690,000 new annual cases of cancer in females and males globally, including cancers of the cervix, head and neck, anus, penis, vagina, and vulva (based on 2018 estimates [1]). HPV types 16 and 18 account for approximately 72% of all HPV-related cases, and types 31, 33, 45, 52, and 58 account for an additional 17% (1). In addition, the low-risk HPV types 6 and 11 are responsible for approximately 90% of all cases of genital warts (2). The available prophylactic vaccines with which to prevent HPV-related infection and disease include the quadrivalent and 9-valent (9vHPV) HPV vaccines, which protect against HPV types 6, 11, 16, and 18, and the 9vHPV vaccine also protects against HPV types 31, 33, 45, 52, and 58 (3). Both vaccines are composed of virus-like particles (VLPs) that are made via the expression of the L1 major capsid protein of each HPV type in eukaryotic cells. Following a demonstration of their efficacy and immunogenicity in clinical trials, the quadrivalent HPV and 9vHPV vaccines have been marketed since 2006 and 2014, respectively. Two bivalent prophylactic vaccines targeting HPV types 16 and 18 are also available, including the AS04-adjuvanted vaccine (marketed since 2007) and an aluminum-adjuvanted vaccine (marketed in China since 2019) (3, 4).

The need to monitor HPV antibody responses in vaccine clinical trials and epidemiological studies has led to the development of various immunoassays, such as neutralization and pseudoneutralization assays, epitope-specific competitive immunoassays, and type-specific direct binding immunoglobulin G (IgG) assays (5–15). Since developing neutralization assays for HPV would be challenging, given the difficulties in growing HPV *in vitro*, pseudoneutralization assays for VLP L1 and L2 and other surrogate assays have been developed to measure functional HPV antibody responses (11–13). However, because pseudoneutralization assays are labor-intensive and technically complex, they are not ideal for high-throughput testing (16). Epitope-specific competitive immunoassays use neutralizing monoclonal antibodies that bind to conformational L1 epitopes to measure type-specific antibodies that recognize dominant neutralizing epitopes of VLPs (6–8, 14–16). These types of assays are useful as surrogates for neutralization assays, and they have the benefit of being type-specific and sensitive. The caveats of epitope-specific competitive immunoassays include: (i) that they only measure a subset of the total anti-VLP antibodies and (ii) that they only measure a single neutralizing epitope. Thus, the total levels of protective antibodies may potentially be underrepresented. Type-specific direct binding IgG assays measure the total levels of all HPV-type-specific binding IgG antibodies, including both functional and nonfunctional antibodies (9, 10, 16). These assays are sensitive, reproducible, and suitable for high-throughput testing.

The 9vHPV vaccine clinical trial program has relied on 2 multiplexed immunoassays to assess anti-HPV antibody responses, and versions of these assays continue to be used in ongoing trials. These immunoassays are the HPV6/11/16/18/31/33/45/52/58 competitive Luminex immunoassay (HPV-9 cLIA), an epitope-specific competitive immunoassay, and the HPV6/11/16/18/31/33/45/52/58 total IgG Luminex immunoassay (HPV-9 IgG-LIA), which is a type-specific direct binding IgG assay. Both the HPV-9 cLIA and HPV-9 IgG-LIA are Luminex immunoassays involving type-specific, yeast-derived VLPs that are coupled to 9 distinct Luminex microspheres, called VLP-coupled microspheres or MS-VLP. Each microsphere has its own distinct fluorescent dye that can be identified following excitation with an infrared laser, which allows for the measurement of multiple HPV types from a single test of a human serum sample.

The original versions of the HPV-9 cLIA and HPV-9 IgG-LIA that have been used in clinical trials through 2015 have been described previously (7, 9, 15). As described herein, both the HPV-9 cLIA and HPV-9 IgG-LIA were redeveloped, and the optimized assays have been validated and reviewed by the Center for Biologics Evaluation and Research within the U.S. Food and Drug Administration. The reoptimized versions have been used for the testing of clinical trial samples since 2016. The new versions of the assays were bridged to the previous versions, and serostatus cutoffs (SSCOs) in the new versions were established so as to seamlessly continue assessments of antibody responses and seropositivity rates in ongoing and future studies. We describe the redevelopment, validation, and SSCO assignments for the new versions of the HPV-9 cLIA and HPV-9 IgG-LIA.

## RESULTS

### Optimization of reagents used in the HPV-9 cLIA and IgG-LIA.

The optimal VLP coupling concentration was determined to be 100 μg/mL for HPV11, 16, 18, 31, 33, 45, 52, and 58 and 200 μg/mL for HPV6, based on the VLP coating saturation points. The optimal VLP coupling concentrations were selected, based on maximizing the dynamic range of the median fluorescence intensity values that were observed over a range of concentrations that were evaluated. Following a comparison with 2 ready-to-use wash buffers, the original 1× phosphate-buffered saline (PBS) + 0.05% Triton X-100 buffer was found to be equivalent to both the 1× PBS Tween 20 and 1× Focus Wash buffers. The 1× PBS Tween 20 wash buffer was selected, given that preweighed packets may be stored at ambient temperature and that the buffer may be prepared by adding distilled water.

### Redevelopment and optimization of the HPV-9 cLIA and IgG-LIA.

The significant parameters that were reoptimized for the HPV-9 cLIA and HPV-9 IgG-LIA are summarized in Table 1, including the use of magnetic Luminex microspheres, elimination of antibody-depleted human serum (ADHS) from the diluent, changing the source of the phycoerythrin (PE)-conjugates to improve lot-to-lot consistency, and changing the reference standard and serum sample starting dilution for the HPV-9 cLIA. Following initial evaluation of the reference standard starting dilution for the HPV-9 IgG-LIA assay, a 1:100 starting dilution was selected with a 2.1-fold titration to achieve the optimal dynamic range. In addition, the working concentration of 2.5 μg/mL for the Jackson conjugate was selected for the HPV-9 IgG-LIA assay.

**TABLE 1:** Summary of significant parameters reoptimized for HPV-9 cLIA and HPV-9 IgG-LIA^*a*^

Parameter	Previous	Optimized	Conclusion
*HPV-9 cLIA*			
Luminex microspheres	Carboxylated polystyrene	Magnetic	Operational advantage when coupling to VLP; improve washing efficiency
Serum sample and reference standard diluent	ADHS + 0.01% sodium azide	PBS + 1% BSA + 0.05% sodium azide	Decrease dilutional bias from ADHS
Serum sample starting dilution	1:4 and 1:40 simultaneously	1:10	Increase throughput
Reference standard starting dilution	1:20	1:10	Increase no. of standard curve points in the quantifiable range
Reference standard titration series	2-fold serial dilutions	1.7-fold serial dilutions	Increase no. of standard curve points in the quantifiable range
PE-mAb vendor	Chromaprobe	Southern Biotech	Improve lot-to-lot consistency
*HPV-9 IgG-LIA*			
Luminex microspheres	Carboxylated polystyrene	Magnetic	Operational advantage when coupling to VLP; improve washing efficiency
Serum sample starting dilution	1:100 and 1:10,000 simultaneously	1:100	Increase throughput
Reference standard starting dilution	1:200	1:100	Increase no. of standard curve points in the quantifiable range
Reference standard titration series	3-fold serial dilutions	2.1-fold serial dilutions	Increase no. of standard curve points in the quantifiable range
PE-labeled goat anti-human IgG vendor	Chromaprobe	Jackson ImmunoResearch Laboratories	Improve lot-to-lot consistency

a ADHS, antibody-depleted human serum; BSA, bovine serum albumin; HPV, human papillomavirus; HPV-9 cLIA, HPV6/11/16/18/31/33/45/52/58 competitive Luminex immunoassay; HPV-9 IgG-LIA, HPV6/11/16/18/31/33/45/52/58 total IgG Luminex immunoassay; IgG, immunoglobulin G; mAb, monoclonal antibody; PBS, phosphate-buffered saline; PE, phycoerythrin; VLP, virus-like particle.

### Validation of the redeveloped HPV-9 cLIA and IgG-LIA.

The assay validation data for the HPV-9 cLIA and IgG-LIA are summarized in Table 2 and Table S1, and the results of the intermediate precision (incurred and dilution linearity samples), linearity, and relative accuracy analyses are described below. Overall, all of the acceptance criteria for assay validation were met (Table S1), indicating that the assays are accurate, specific, and precise throughout their quantifiable ranges for each HPV type.

**TABLE 2:** Summary of key validation results for the HPV-9 cLIA and HPV-9 IgG-LIA^*a*^

Parameter	Results	
	HPV-9 cLIA	HPV-9 IgG-LIA
Intra-assay precision (repeatability)	Overall %RSD < 10% for each HPV type	Overall %RSD < 4% for each HPV type
Interassay precision (incurred samples)	Overall %RSD < 11% for each HPV type 100% of samples within the quantifiable range (176/176) had %RSD < 20%	Overall %RSD < 8% for each HPV type 100% of samples within the quantifiable range (178/178) had %RSD < 12%
Interassay precision (dilution linearity samples)	Overall %RSD < 9% for each HPV type 95.2% of samples within the quantifiable range (299/314) had %RSD < 25%	Overall %RSD < 8% for each HPV type 100% of samples within the quantifiable range (375/375) had %RSD < 20%
Linearity	Average slope within a range of −1.25 and −0.80 for each sample and each HPV type	Average slope within a range of −1.20 and −0.90 for each sample and each HPV type
Relative accuracy	Overall estimate of dilution bias within 1.25-fold per 10-fold dilution	Overall estimate of dilution bias within 1.25-fold per 10-fold dilution

a %RSD, percent relative standard deviation; HPV, human papillomavirus; HPV-9 cLIA, HPV6/11/16/18/31/33/45/52/58 competitive Luminex immunoassay; HPV-9 IgG-LIA, HPV6/11/16/18/31/33/45/52/58 total IgG Luminex immunoassay; IgG, immunoglobulin G.

10.1128/msphere.00962-21.1 TABLE S1 Summary of validation parameters for the new versions of the HPV-9 cLIA and IgG-LIA. *^a^*Acceptance limits were based on: (i) experience with respect to acceptable performance for ligand binding assays in general and (ii) the historical performance of the original versions of the HPV-9 cLIA and HPV-9 IgG-LIA. %RSD, percent relative standard deviation; cLIA, competitive Luminex immunoassay; HPV, human papillomavirus; HPV-9 cLIA, HPV6/11/16/18/31/33/45/52/58 cLIA; HPV-9 IgG-LIA, HPV6/11/16/18/31/33/45/52/58 total IgG Luminex immunoassay; IgG, immunoglobulin G; LLOQ, lower limit of quantitation; mAb, monoclonal antibody; mMU, milli-Merck Units; MS-VLP, VLP-coupled microspheres; PE, phycoerythrin; RMSE, root mean square error; SSCO, serostatus cutoff; ULOQ, upper limit of quantitation; VLP, virus-like particle. Download Table S1, DOCX file, 0.02 MB. Copyright © 2023 Nolan et al. 2023 Nolan et al. https://creativecommons.org/licenses/by/4.0/ This content is distributed under the terms of the Creative Commons Attribution 4.0 International license.

**(i) HPV-9 cLIA precision, linearity, and relative accuracy.** In the analysis of interassay precision, which involved a panel of 20 (incurred) human serum samples (each with 12 replicates) from individuals who were vaccinated for all 9 HPV types, the percent relative standard deviation (%RSD) was <20% for all samples with antibody concentrations within the quantifiable range of the assay. The overall precision was <11% for each HPV type. As shown in Table 2, similar precision estimates were obtained for the dilution linearity sample set. On average, the ratios of antibody concentrations using different PE-labeled monoclonal antibody (mAb) and MS-VLP lots were within ±1.03-fold and 1.04-fold, respectively, for each HPV type.

In the analysis of linearity, which involved 72 individual test sample dilution curves per HPV type, the slope average was within the range of −1.25 to −0.80 for each sample and HPV type. The R^2^ value was >0.97 for all 648 of the individual dilution curves.

For each HPV type, the overall estimate of dilution bias was within 1.25-fold per 10-fold dilution. Of the 648 individual estimates of dilution bias that were obtained across the set of runs, samples, and HPV types, 646 were ≤2.0-fold per 10-fold dilution.

**(ii) HPV-9 IgG-LIA precision, linearity, and relative accuracy.** In the analysis of interassay precision, which involved a panel of 20 (incurred) human serum samples (each with 12 replicates) from individuals vaccinated for all 9 HPV types, all samples with antibody concentrations within the quantifiable range of the assay had %RSD < 12%. The overall precision was <8% for each HPV type. As shown in Table 2, similar precision estimates were obtained for the dilution linearity sample set. On average, the ratios of IgG concentrations using different IgG-PE and MS-VLP lots were within ±1.03 for each HPV type.

In the analysis of linearity, which involved 71 individual test sample dilution curves per HPV type, the slope was within the range of −1.20 to −0.90 for each curve and HPV type. The R^2^ value was >0.99 for all 639 of the individual dilution curves.

All 639 of the individual estimates of dilution bias obtained across the set of runs, samples, and HPV types were ≤2.0-fold per 10-fold dilution. The observed dilution bias was similar across the set of test samples and HPV types, as the concentrations corrected for dilution tended to decrease slightly with increasing dilution. The mean estimated bias was −17.4% to −15.2% per 10-fold dilution across each HPV type.

### Bridging to previous versions of the assays and SSCO determinations.

**(i) HPV-9 cLIA bridging.** Bridging studies were performed to allow for comparison of the results from the optimized assays to historical results obtained using the previous assays. The HPV-type-specific lower limits of quantitation (LLOQs) and upper limits of quantitation (ULOQs) for the new versus previous HPV-9 cLIAs are summarized in Table 3.

**TABLE 3:** Limits of quantitation and SSCOs (mMU/mL) for the optimized versus previous versions of the HPV-9 cLIA and IgG-LIA^*a*^

Version	LLOQ	Optimized			Previous	
		ULOQ	SSCO	LLOQ	ULOQ	SSCO
*HPV-9 cLIA*
HPV6	20	1,005	50	16	171	30
HPV11	16	664	29	6	113	16
HPV16	20	3,581	41	12	609	20
HPV18	24	956	59	8	163	24
HPV31	10	875	29	4	149	10
HPV33	8	474	22	4	81	8
HPV45	8	351	15	3	60	8
HPV52	8	432	20	3	73	8
HPV58	8	546	15	4	93	8
*HPV-9 IgG-LIA*
HPV6	2	500	9	2	570	15
HPV11	2	300	6	3	380	15
HPV16	4	1,700	5	5	2,030	7
HPV18	3	540	5	10	540	10
HPV31	2	400	3	2	500	6
HPV33	2	270	4	3	270	6
HPV45	1	200	3	17	200	17
HPV52	1	200	5	4	240	8
HPV58	2	250	5	4	310	6

a HPV, human papillomavirus; HPV-9 cLIA, HPV6/11/16/18/31/33/45/52/58 competitive Luminex immunoassay; HPV-9 IgG-LIA, HPV6/11/16/18/31/33/45/52/58 total IgG Luminex immunoassay; IgG, immunoglobulin G; LLOQ, lower limit of quantitation; mMU, milli-Merck Units; SSCO, serostatus cutoff; ULOQ, upper limit of quantitation.

**FIG 1:**
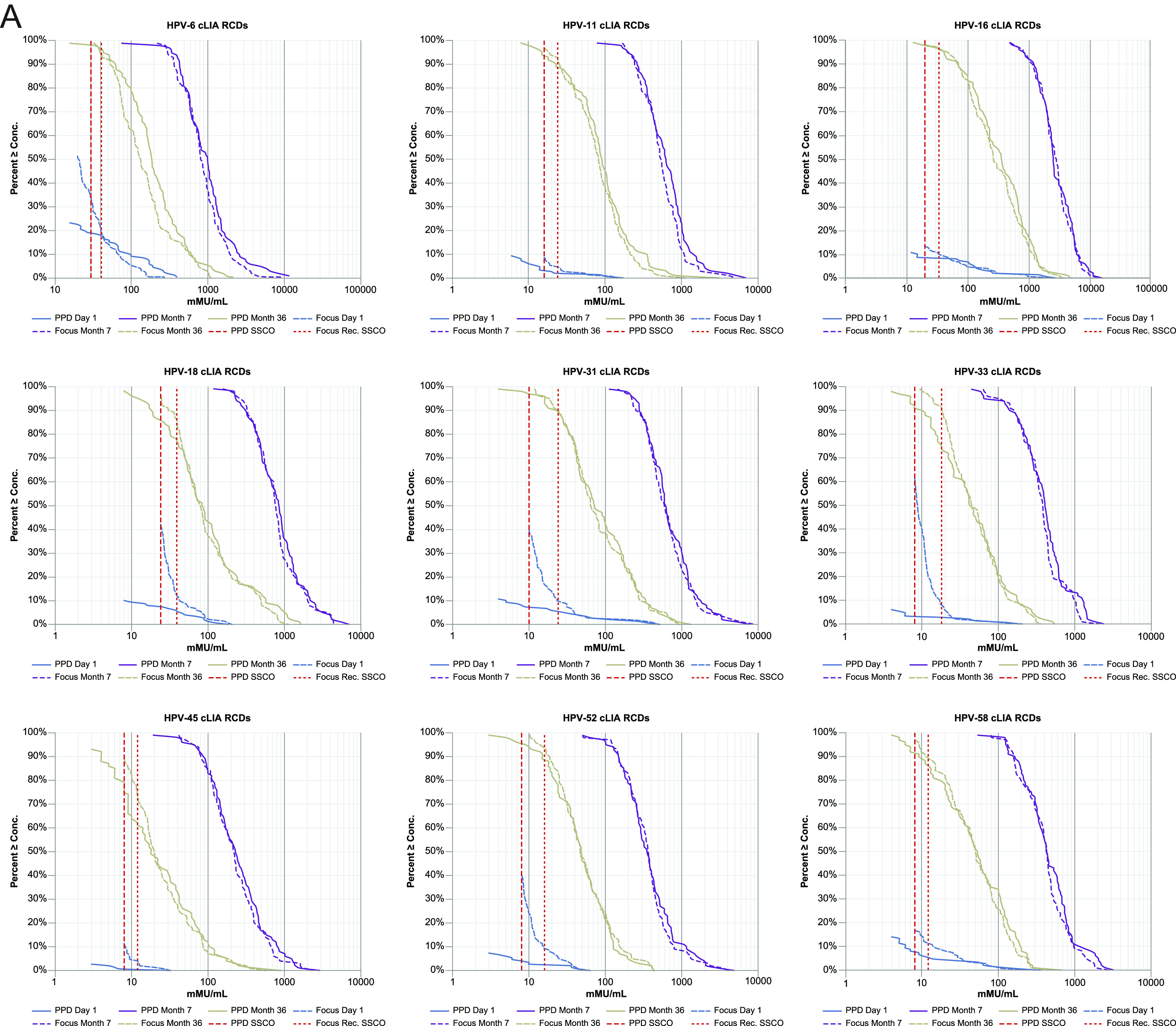

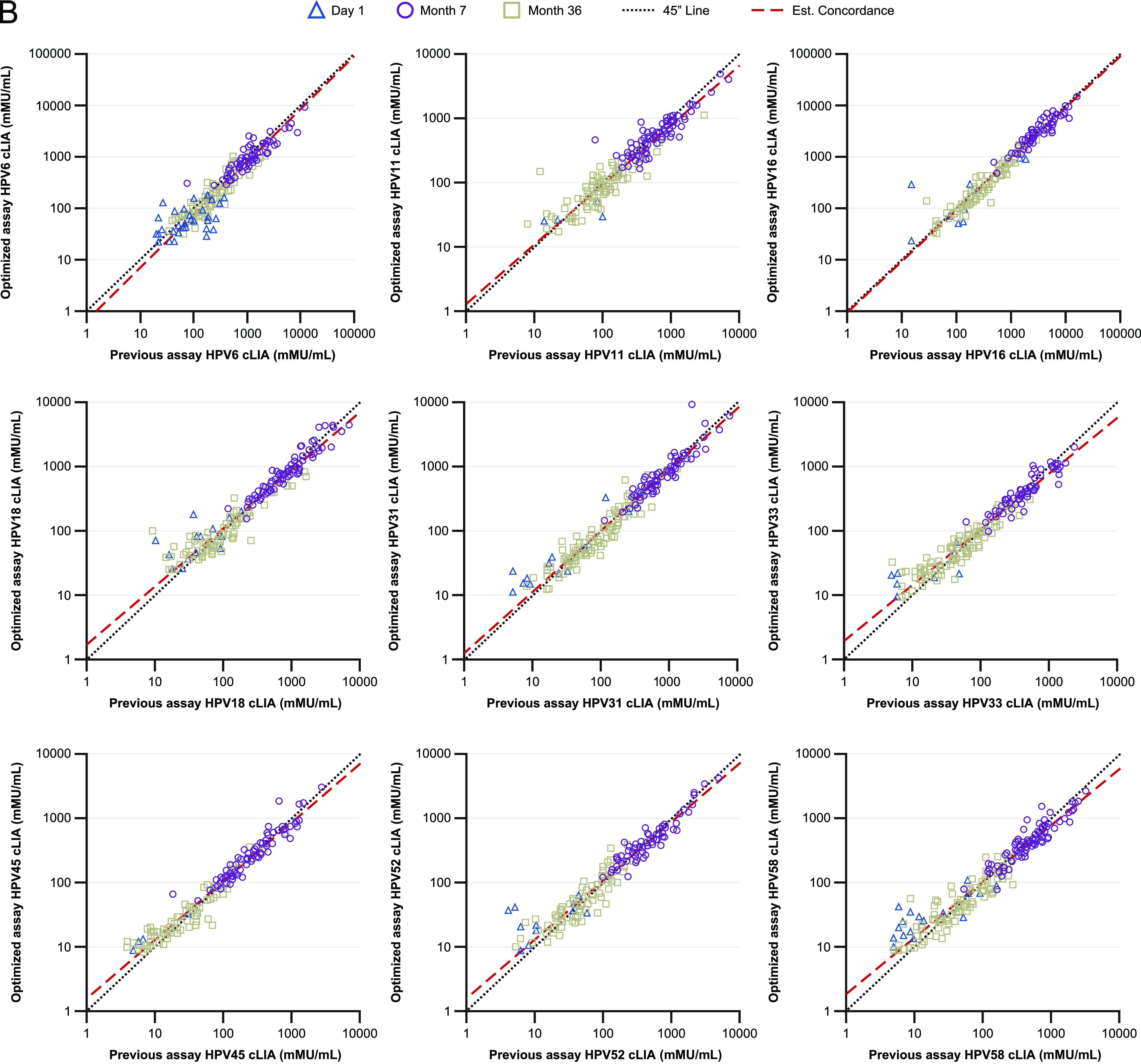
Reverse cumulative distribution plots (A) and HPV concordance plots (B), comparing the HPV-9 cLIA versions. cLIA, competitive Luminex immunoassay; HPV, human papillomavirus; HPV-9 cLIA, HPV6/11/16/18/31/33/45/52/58 cLIA; mMU, milli-Merck Units; RCD, reverse cumulative distribution; SSCO, serostatus cutoff.

For each of the 9 HPV types, the reverse cumulative distribution (RCD) curves for the 2 assay versions overlay one another closely for the month 7 and month 36 postvaccination samples (Fig. 1A). The assay versions were more divergent for the day 1 samples, with concentrations being close to the assay SSCOs. The detected concentrations for samples with low but detectable antibody levels (“low-positive samples”), as defined by the SSCO of the previous assay version, tended to be higher in the new version, compared with the previous version of the assay, suggesting that the SSCO in the new cLIA should be increased, relative to the previous version, to yield a similar day 1 positivity rate. For each HPV type, there was a strong, positive, linear association between the new and previous cLIA versions (Fig. 1B). For samples having a determinate concentration in both assay versions, the fitted concordance slope across the 9 HPV types ranged between 0.86 (for HPV33) to 1.03 (for HPV6) (Table 4). The geometric mean fold-difference across the 9 HPV types ranged between 0.79 (for HPV6) and 1.07 (for HPV52). In addition, the Pearson correlation coefficient and Lin’s concordance correlation coefficient exceeded 0.934 and 0.919, respectively, across the 9 HPV types.

**TABLE 4:** Concordance estimates between the optimized and previous HPV-9 cLIA and IgG-LIA versions by HPV type^*a*^

Version	Slope estimate (95% CI)	Intercept estimate (95% CI)	Geometric mean fold difference estimate (95% CI)	Coefficient		
				Pearson correlation	Lin’s accuracy	Lin’s correlation
*HPV-9 cLIA*						
HPV6	1.03 (0.98, 1.08)	−0.39 (−0.45, −0.33)	0.79 (0.75, 0.84)	0.934	0.983	0.919
HPV11	0.93 (0.88, 0.98)	0.26 (0.21, 0.32)	0.88 (0.83, 0.94)	0.942	0.993	0.935
HPV16	1.00 (0.97, 1.04)	−0.08 (−0.13, −0.03)	0.93 (0.89, 0.98)	0.969	0.999	0.968
HPV18	0.90 (0.87, 0.94)	0.57 (0.52, 0.62)	1.01 (0.95, 1.07)	0.961	0.995	0.956
HPV31	0.95 (0.92, 0.98)	0.26 (0.22, 0.31)	0.98 (0.93, 1.03)	0.971	0.998	0.970
HPV33	0.86 (0.83, 0.89)	0.69 (0.65, 0.74)	1.04 (0.98, 1.09)	0.968	0.990	0.958
HPV45	0.90 (0.87, 0.93)	0.45 (0.41, 0.50)	1.00 (0.95, 1.05)	0.974	0.995	0.969
HPV52	0.90 (0.87, 0.94)	0.54 (0.49, 0.59)	1.07 (1.01, 1.13)	0.961	0.994	0.955
HPV58	0.88 (0.84, 0.91)	0.62 (0.57, 0.67)	1.01 (0.95, 1.08)	0.960	0.992	0.952
*HPV-9 IgG-LIA*						
HPV6	1.07 (1.04, 1.09)	−0.54 (−0.58, −0.49)	0.81 (0.77, 0.85)	0.981	0.992	0.973
HPV11	1.10 (1.06, 1.13)	−0.63 (−0.67, −0.58)	0.85 (0.81, 0.90)	0.970	0.990	0.961
HPV16	1.03 (1.00, 1.06)	−0.34 (−0.39, −0.30)	0.86 (0.83, 0.90)	0.981	0.996	0.977
HPV18	1.09 (1.04, 1.13)	−0.64 (−0.71, −0.58)	0.83 (0.78, 0.89)	0.960	0.990	0.950
HPV31	1.02 (0.98, 1.07)	−0.28 (−0.34, −0.21)	0.86 (0.81, 0.91)	0.958	0.995	0.953
HPV33	1.02 (0.98, 1.06)	−0.16 (−0.21, −0.11)	0.92 (0.87, 0.97)	0.961	0.998	0.959
HPV45	1.13 (1.00, 1.28)	−0.95 (−1.08, −0.80)	0.73 (0.64, 0.83)	0.789	0.966	0.762
HPV52	1.00 (0.95, 1.04)	−0.13 (−0.19, −0.07)	0.86 (0.81, 0.91)	0.952	0.994	0.947
HPV58	1.04 (1.00, 1.07)	−0.32 (−0.37, −0.27)	0.85 (0.81, 0.90)	0.966	0.994	0.960

a CI, confidence interval; HPV, human papillomavirus; HPV-9 cLIA, HPV6/11/16/18/31/33/45/52/58 competitive Luminex immunoassay; HPV-9 IgG-LIA, HPV6/11/16/18/31/33/45/52/58 total IgG Luminex immunoassay; IgG, immunoglobulin G.

**(ii) HPV-9 cLIA SSCO determination.** SSCOs for the reoptimized HPV-9 cLIA were initially established by testing the individual participant serum from Study V503-001 in the bridging study and comparing the results with those obtained from the previous cLIA for the same samples. For each HPV type, the SSCO was determined by balancing the discrepancy between the assay versions in the day 1 and month 36 positivity rates, with greater emphasis being placed on aligning the assays on the day 1 negativity rate. SSCOs were assigned to achieve positivity rates for the day 1 and month 36 samples that were comparable to the corresponding rates from the earlier version of the assay.

The monitoring of HPV-9 cLIA data from ongoing testing of clinical study samples indicated that further SSCO refinement was needed to achieve similar rates of HPV18 baseline positivity between the previous and reoptimized cLIAs. When early serum samples from adult female trial participants were measured using the updated cLIA, the baseline positivity rates for HPV18 (41.4%) substantially exceeded the historical HPV18 baseline positivity rates in adult women as well as those rates predicted by the laboratory bridging study (12.7%), whereas the observed baseline positivity rates for the 8 other HPV types either closely approximated or slightly exceeded the expected positivity rates, based on the results of the bridging study. The RCD curves of the anti-HPV18 antibody concentrations in the clinical study baseline samples showed that the vast majority of samples (approximately 87.5%) had antibody concentrations below 59 milli-Merck Units (mMU)/mL in adult female clinical trial participants (Fig. S1). Similar findings were observed across the baseline samples that were tested using the revised HPV-9 cLIA across active clinical studies. An investigation into the elevated baseline positivity rate for HPV18 ensued. The most likely cause for the unexpectedly high anti-HPV18 baseline positivity rate was that the HPV18 cLIA appears to have been running low by a factor of 1.25-fold in the region of the SSCO at the time that the bridging data were generated, as evidenced by the performance of the quality-control samples during that period. Given that the originally recommended SSCO for HPV18 was likely underestimated by a factor of 1.25-fold, a 1.25-fold increase in the SSCO for HPV18 was recommended. The investigation also brought attention to the bridging study result that the redeveloped cLIA had a higher proportion of samples in close proximity to the SSCO, compared to the previous cLIA version (Fig. 1A). The consequence of this is that even minor movement in an assay that may occur over an extended time, particularly at its low end, had the potential to meaningfully impact the positivity rate in the redeveloped cLIA. Therefore, to mitigate the potential for any of the HPV types to have a similarly inflated baseline positivity rate in the redeveloped cLIA, to maintain bridging with the historical baseline seropositivity rates obtained using the previous assay version, and to have minimal impact on antibody persistence rates at later time points (≥36 months), the SSCOs for each of the 9 HPV types were increased by 1.2-fold (Table 3). The refined SSCO for HPV18 resulted in a baseline positivity rate that was consistent with that of the original laboratory bridging study and better aligned with the rates for the 8 other HPV types.

10.1128/msphere.00962-21.2 FIG S1 HPV18 RCD curves for the baseline samples from the adult female clinical trial participants, using the updated HPV-9 cLIA. HPV, human papillomavirus; HPV-9 cLIA, HPV6/11/16/18/31/33/45/52/58 competitive Luminex immunoassay; mMU, milli-Merck Units; RCD, reverse cumulative distribution. Download FIG S1, EPS file, 1.4 MB. Copyright © 2023 Nolan et al. 2023 Nolan et al. https://creativecommons.org/licenses/by/4.0/ This content is distributed under the terms of the Creative Commons Attribution 4.0 International license.

**FIG 2:**
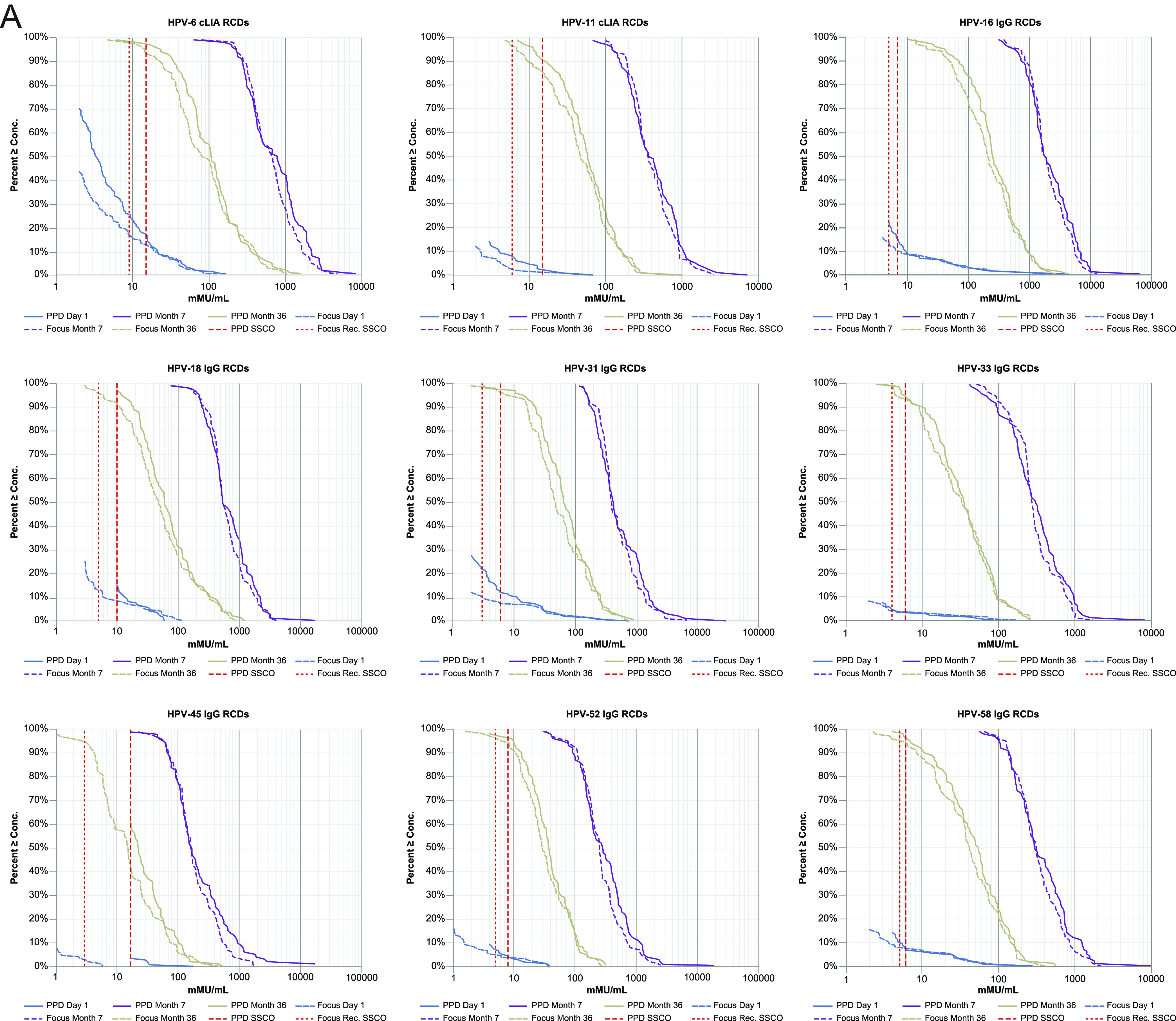

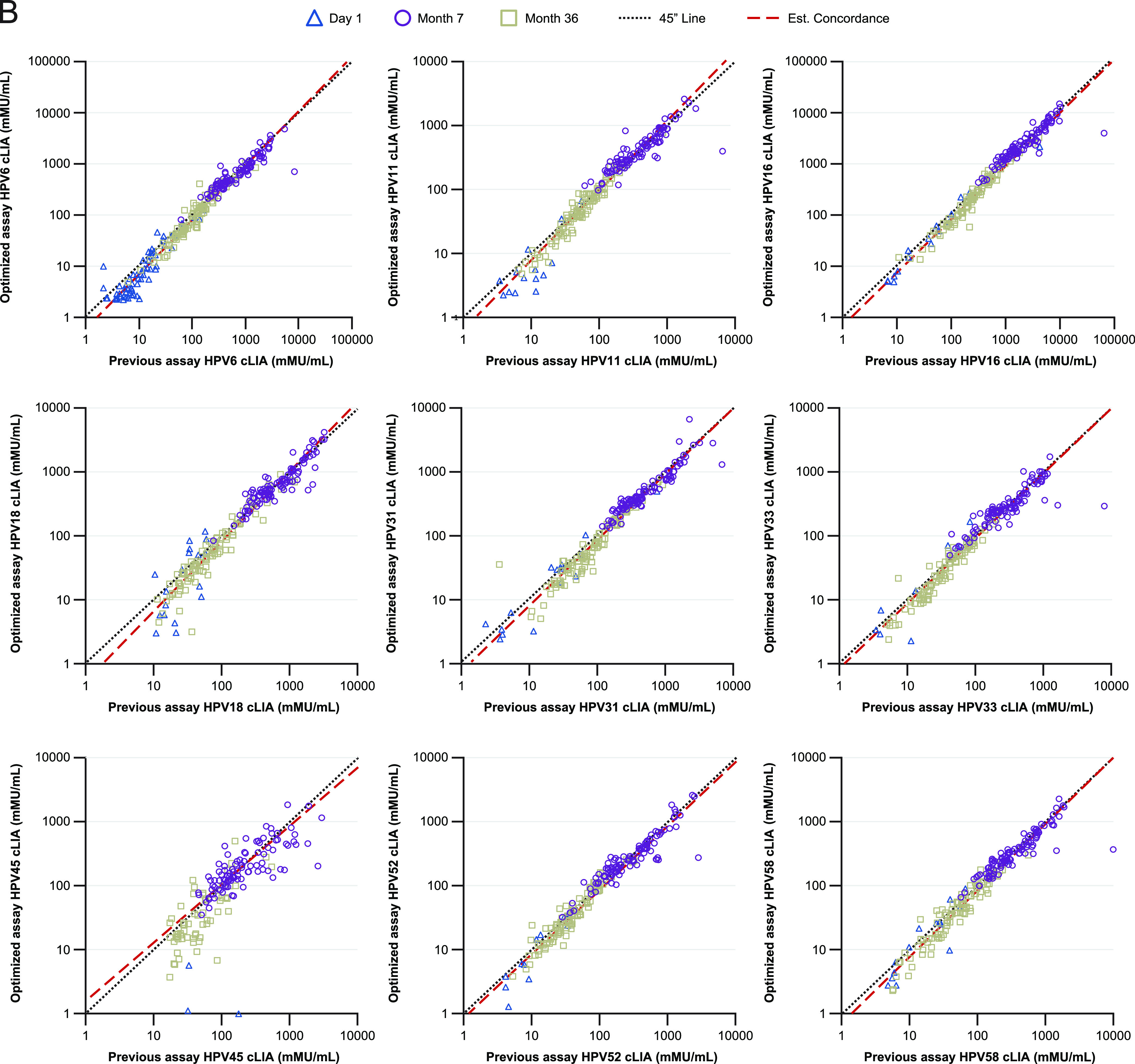
Reverse cumulative distribution plots (A) and HPV concordance plots (B), comparing the HPV-9 IgG-LIA versions. HPV, human papillomavirus; HPV-9 IgG-LIA, HPV6/11/16/18/31/33/45/52/58 total IgG Luminex immunoassay; IgG, immunoglobulin G; mMU, milli-Merck Units; RCD, reverse cumulative distribution; SSCO, serostatus cutoff.

**(iii) HPV-9 IgG-LIA bridging.** The HPV-type-specific LLOQs and ULOQs for the new versus previous HPV-9 IgG-LIAs are summarized in Table 3. For each of the 9 HPV types, the RCD curves for the 2 assay versions overlay one another closely for the month 7 post-vaccination samples (Fig. 2A). For HPV types 6, 11, 18, and 45, the RCD curves revealed some divergence between the assay versions for the day 1 and month 36 samples. Concentration measures for the low-positive samples tended to be lower in the new assay version, compared with the previous version, suggesting that the SSCO in the new version needed to be slightly reduced, relative to the previous assay, to yield a similar day 1 positivity rate. For each HPV type, there was a strong positive linear association between the new and previous IgG-LIA versions (Fig. 2B). Among samples with a determinate concentration in both assay versions, the fitted concordance slope across the 9 HPV types ranged between 1.00 for HPV52 to 1.13 for HPV45 (Table 4). The geometric mean fold-difference across the 9 HPV types ranged between 0.81 (for HPV6) and 0.92 (for HPV33). Additionally, the Pearson correlation coefficient and Lin’s concordance correlation coefficient exceeded 0.952 and 0.947, respectively, across 8 of the 9 HPV types (excluding HPV45). For HPV45, the Pearson correlation coefficient and Lin’s concordance correlation coefficient were 0.789 and 0.762, respectively. The weaker interassay correlation for HPV45 is attributed to the performance of the previous version of the assay, as evidenced by the difference in the LLOQ values between the assay versions (17 mMU/mL versus 1 mMU/mL for the previous and new IgG-LIA, respectively).

**(iv) HPV-9 IgG-LIA SSCO determination.** Based on the selection criteria that are described in Materials and Methods, the SSCOs for the new HPV-9 IgG-LIA were defined (Table 3). For HPV45, the updated SSCO resulted in a marked improvement in sensitivity for the month 36 samples, compared with the previous version of the assay. For the other 8 HPV types, the assay sensitivity for the new version of the IgG-LIA was similar to or slightly improved, compared with that of the previous version, and the SSCOs for the new version of the IgG-LIA were similar or slightly reduced, relative to those of the previous version.

### HPV-16 and HPV-18 reference standard evaluation.

International reference standards containing HPV-16 and HPV-18 specific antibodies were analyzed in both the cLIA and IgG-LIA assays to demonstrate the relative performance of the Merck & Co., Inc., Rahway, NJ, USA (MSD) internal reference standard and corresponding concentration units (mMU/mL). The resulting measured antibody concentrations in the cLIA and IgG-LIA for the HPV-16 and HPV-18 international reference standards are listed in Table 5. Consistently quantifiable results were obtained in the cLIA at the 1:10 and 1:15 dilutions for the HPV-16 international reference standard as well as at the 1:10, 1:15, and 1:22.5 dilutions for the HPV-18 international reference standard. Consistently quantifiable results were obtained in the IgG-LIA at all six of the evaluated dilutions for both international reference standards. For both the cLIA and IgG-LIA, the dilution-corrected measured concentrations were consistent across the evaluable dilutions, indicating that the international standards dilute in parallel to the MSD reference standard in their respective assays.

**TABLE 5:** Evaluation of HPV-16 and HPV-18 International Reference Standards^*a*^

Assay	HPV type	Dilution	Day 1				Day 2				Day 3				Mean concn mMU/mL		IU/mL equivalence	
			Plate 1		Plate 2		Plate 3		Plate 4		Plate 5		Plate 6				To 1 mMU/mL	
			1	2	1	2	1	2	1	2	1	2	1	2	Estimate	95% CI	Estimate	95% CI
cLIA	HPV-16	1:10	93	85	89	97	88	113	109	114	98	112	93	99	99.2	(88.8, 109.5)	0.101	(0.091, 0.113)
cLIA	HPV-16	1:15	88	84	85	103	106	112	123	105	95	110	99	104	101.2	(83.9, 118.4)	0.099	(0.084, 0.119)
cLIA	HPV-18	1:10	123	118	121	122	153	137	138	136	119	121	119	114	126.8	(95.9, 157.6)	0.126	(0.102, 0.167)
cLIA	HPV-18	1:15	126	117	128	112	166	133	148	130	126	117	123	119	128.8	(108.8, 148.7)	0.124	(0.108, 0.147)
cLIA	HPV-18	1:22.5	130	92	123	114	162	143	132	137	126	111	125	121	126.3	(103.8, 148.8)	0.127	(0.107, 0.154)
IgG	HPV-16	1:100	97	91	90	90	83	90	83	90	93	92	92	88	89.9	(85.9, 94)	0.111	(0.106, 0.116)
IgG	HPV-16	1:150	94	95	94	96	94	93	87	91	96	97	98	89	93.7	(90.9, 96.5)	0.107	(0.104, 0.11)
IgG	HPV-16	1:225	98	95	91	94	98	92	87	93	98	97	99	90	94.3	(91.6, 97.1)	0.106	(0.103, 0.109)
IgG	HPV-16	1:337.5	95	97	90	95	99	91	88	96	98	99	101	85	94.5	(91, 98)	0.106	(0.102, 0.11)
IgG	HPV-16	1:506.25	95	99	91	97	97	92	88	97	99	103	101	87	95.5	(91.9, 99.1)	0.105	(0.101, 0.109)
IgG	HPV-16	1:759.375	92	100	90	94	98	90	89	101	100	103	98	85	95.0	(90.9, 99.1)	0.105	(0.101, 0.11)
IgG	HPV-18	1:100	80	95	90	75	84	79	85	82	87	85	102	92	86.3	(80, 92.7)	0.185	(0.173, 0.2)
IgG	HPV-18	1:150	89	97	88	87	86	91	86	87	88	89	105	94	90.6	(86, 95.2)	0.177	(0.168, 0.186)
IgG	HPV-18	1:225	86	94	85	86	84	89	86	85	91	88	106	94	89.5	(83.5, 95.5)	0.179	(0.168, 0.192)
IgG	HPV-18	1:337.5	83	92	84	83	82	90	85	83	86	85	107	91	87.6	(82.2, 93)	0.183	(0.172, 0.195)
IgG	HPV-18	1:506.25	82	87	81	83	79	87	85	81	85	83	109	86	85.7	(79.7, 91.7)	0.187	(0.175, 0.201)
IgG	HPV-18	1:759.375	82	82	81	77	79	82	84	78	84	80	111	89	84.1	(76.4, 91.8)	0.190	(0.174, 0.209)

a cLIA, competitive Luminex immunoassay; IgG, immunoglobulin G Luminex immunoassay; HPV-16 human papillomavirus serotype 16; HPV-18, human papillomavirus serotype 18; CI, confidence interval; IU/mL, International Units per milliliter; mMU/mL, milli-Merck Units per milliliter.

Across the evaluable dilutions in the cLIA, the mean concentrations for the HPV-16 and HPV-18 international reference standards in their respective assays were 100.2 and 127.3 mMU/mL, respectively. Given the assigned antibody concentrations for the HPV-16 and HPV-18 international reference standards of 10 IU/mL and 16 IU/mL, respectively, the resulting conversion factor between mMU/mL and IU/mL is 1 mMU/mL, which corresponds to 0.100 IU/mL for the HPV-16 cLIA and 0.126 IU/mL for the HPV-18 cLIA.

Across the six evaluable dilutions in the IgG-LIA, the mean concentrations for the HPV-16 and HPV-18 international reference standards in their respective IgG-LIAs were 93.8 and 87.3 mMU/mL, respectively. Using the assigned antibody concentrations for the HPV-16 and HPV-18 international reference standards, the resulting conversion factor between mMU/mL and IU/mL is 1 mMU/mL, which corresponds to 0.107 IU/mL for the HPV-16 IgG-LIA and 0.183 IU/mL for the HPV-18 IgG-LIA.

## DISCUSSION

Optimized versions of the HPV-9 cLIA and IgG-LIA have been developed and validated for use in high-throughput testing for HPV antibodies in serum samples. The optimized parameters, including the elimination of ADHS in the assay buffer and increasing the serum sample starting dilution (from 1:4 to 1:10), improved the dilutability of the HPV-9 cLIA. Results of the validation studies indicate that both optimized assays are accurate, specific, and precise throughout the assay quantifiable range for each HPV type.

In bridging studies to the prior versions of the HPV-9 cLIA and IgG-LIA, strong positive linear associations between the previous and new assay versions were observed. This allows for continuous immunogenicity assessments in clinical trials of 9vHPV vaccines across assay versions. For example, long-term antibody persistence has been evaluated in long-term follow-up studies (17–19) across the previous and new assay versions. Immunobridging approaches (i.e., demonstrations of noninferior anti-HPV immune responses, compared with a benchmark cohort of young women [aged 16 to 26 years] in whom efficacy has been established) have been used throughout the 9vHPV vaccine clinical development program to extrapolate vaccine efficacy to additional demographic groups (20–23). Consistency across immunoassay versions is required for additional immunobridging studies that are conducted to expand licensure to additional populations and dosing regimens.

SSCOs have been established to identify people with serologic response to vaccination. For both optimized assays, SSCOs were initially defined based on the bridging studies, compared with the prior assay versions, using human serum samples from a single clinical study. In the case of the cLIA, the SSCO values were further refined, based on an observed, higher-than-expected HPV18 baseline positivity in actual clinical trial samples from additional studies that were conducted early after the assay reoptimization. This result highlights the importance of including relevant clinical sample panels for SSCO evaluation. Since biological assays, such as cLIA and LIA, are expected to evolve over time as instrumentation and reagents are updated or modernized, cLIA and IgG-LIA performance will continue to be monitored across ongoing and future clinical studies so as to assess the potential need for modification to provide a sustainable immunogenicity endpoint and to enable immunobridging to historical studies.

Including assay modernization as a key part of vaccine life cycle management has intrinsic benefits, including increased method robustness, cost reduction, and decreased risk of assay failure (24). Vaccine assays, such as the HPV-9 cLIA assay, are a key part of vaccine development and life cycle management, and they may help to extend the overall vaccine life cycle by bridging clinical findings across trials, thereby lengthening the time on patent (15, 20–23, 25). Because vaccine assays may be used for many years beyond the initial clinical studies for which they were developed, assay modernization is important to ensure that they continue to be fit for purpose throughout the vaccine life cycle. For example, assays may be critical for assessing immunogenicity during vaccine coadministration trials, which could take place long after a vaccine has become part of national vaccination programs. Such trials assess the feasibility of concomitantly administering two different vaccines at the same visit, with the aim of maximizing vaccine coverage by administering a newer vaccine with a vaccine that is already part of an existing vaccination program (26–28). In cases of vaccine coadministration trials, it is important to have modernized assays that are capable of reproducibly delineating the immunogenicity associated with each respective vaccine so as to assure effectiveness and the lack of immune interference within target populations.

In summary, the HPV-9 cLIA and IgG-LIA have been reoptimized, validated, and bridged to prior versions of the respective assays for use in ongoing and future HPV vaccine clinical trials.

## MATERIALS AND METHODS

### HPV-9 cLIA and IgG-LIA descriptions.

The HPV-9 (type 6, 11, 16, 18, 31, 33, 45, 52, and 58) immunoassays utilize the Luminex 200 Instrument System, which is a flexible analyzer that is based on the principles of flow cytometry. The basic principles have been described in detail elsewhere (7, 9). Briefly, the same HPV-specific, yeast-derived VLPs that are used in the 9vHPV vaccine are coupled to 9 distinct Luminex microspheres. Each VLP-coupled microsphere has its own distinct fluorescent dye that is recognizable following excitation with an infrared laser, which allows for the measurement of antibodies against multiple HPV types.

The HPV-9 cLIA quantifies antibodies that are specific to dominant neutralizing epitopes. Type-specific, neutralizing monoclonal antibodies are labeled with a PE tag. These tagged antibodies compete with the neutralizing antibodies that are present in the test serum for binding to the corresponding neutralizing epitopes of the VLPs such that the intensity of the fluorescent signal is inversely proportional to the concentration of the anti-HPV neutralizing antibodies. The antibody concentrations are derived from a standard curve that was generated using a reference standard from a pool of serum from individuals immunized against 9 HPV types (the “HPV-9 reference standard”) and calculated using a weighted 4-parameter logistic curve fit. The immune reference serum used as the standard curve was assigned arbitrary values that are expressed in mMU/mL, as previously noted (29). Therefore, the resulting concentrations are expressed in mMU/mL.

The HPV-9 IgG-LIA quantifies the total HPV-type-specific IgG antibodies. VLP-coupled microspheres are incubated with serum and are then washed to remove unbound antibodies. The captured antibodies are subsequently detected using a human IgG antibody-specific PE-conjugated goat antibody. The magnitude of the PE-derived signal is directly proportional to the serum HPV antibody concentration. The antibody concentrations are derived from a standard curve, using the same reference standards as described above, and are expressed in mMU/mL. Although the same term (i.e., mMU/mL) is used for the unit of measurement in both assays, the “HPV-9 cLIA mMU/mL” and the “HPV-9 IgG-LIA mMU/mL” are different units of measurement and cannot be directly compared.

### Assay optimization.

**(i) Optimization of reagents used in both assays.** VLP-coupled beads are prepared using specific bead regions that are selected to minimize overlap between the multiplexed beads. Polystyrene beads, which were used in the previous assay versions, are replaced with MagPlex beads (Luminex Corporation) in the current version of the assays. There are several advantages to MagPlex Microspheres over traditional MicroPlex Microspheres, including their 5× increased multiplex capacity, improved percent recovery during handling and wash steps, and ideal design for automated applications (30). To determine the optimal VLP coating concentrations for each HPV type, a range of VLP concentrations (50 to 500 μg/mL) was examined.

Wash buffer containing Triton X-100 was used in previous assay versions but requires preparation from component reagents. Two ready-to-use wash buffers were compared with the original 1× PBS + 0.05% Triton X-100 wash buffer for suitability for use in the HPV-9 cLIA and IgG-LIA: 1× PBS Tween 20 (prepared from preweighed packets; Sigma) and 1× Focus Wash (diluted from a 10× concentrate of PBS Tween 20; Focus Diagnostics, Cypress, CA).

The HPV-9 reference standard was initially prediluted in antibody-depleted human serum (ADHS) in previous assay versions. The same reference standard and controls were used for the HPV-9 cLIA and HPV-9 IgG-LIA. To minimize the variability observed within the qualified ADHS batches, the use of ADHS was eliminated in the predilution step.

**(ii) Redevelopment of the HPV-9 cLIA.** To optimize the starting concentration and fold dilution of the HPV-9 reference standard (prediluted 1:5 in ADHS for initial optimization; however, upon further optimization, ADHS was eliminated), a 12-point titration curve was created by further dilution of the reference standard to a starting dilution of 1:10. This was followed by 2-fold titration. The serial titration was then adjusted to broaden the range of the standard curve (2-fold, 1.8-fold, and 1.6-fold). A starting dilution of 1:20 was also tested using 2-fold, 1.7-fold, and 1.5-fold titration schemes.

To define the optimal concentrations of the individual PE-labeled mAbs for use in the cLIA, the half maximal effective concentrations (EC_50_) were determined for each PE-mAb, and the reference standard median fluorescence intensity values were compared when testing with PE-mAbs that were prepared at the EC_80_, EC_50_, EC_40_, EC_20_, and EC_10_ concentrations.

To compare the potential sources of the custom conjugated PE-mAbs, PE-conjugated antibodies from three different commercial vendors (Southern Biotech, Birmingham, AL; Columbia Biosciences, Columbia, MD; and Thermo Fisher Scientific [formerly Life Technologies], Waltham, MA) were compared, using HPV16 as a representative HPV-specific mAb. The candidates were tested using 1:5 diluted HPV-9 reference standard and 4 tentative quality-control samples (serum pools from V503-001). The mean interpolated concentrations for HPV16 were calculated, the results from each HPV16 PE-mAb were compared in relation to the expected concentration, and ratios were calculated.

**(iii) Redevelopment of the HPV-9 IgG-LIA.** Reference standard starting dilutions of 1:100, 1:50, 1:40, 1:30, 1:20, and 1:10 were evaluated using a 3-fold titration, and ratios of the observed versus the expected concentrations were calculated for each starting dilution, with the average ratio for each HPV type being used to evaluate the accuracy of the 4-parameter logistic fit. Standard points were considered to be accurate when the ratios were within the range of 0.70 to 1.30.

PE-labeled goat anti-human IgG obtained from Jackson ImmunoResearch Laboratories was compared with the PE-conjugate that was used in the previous version of the assay (HP6043), and the optimal working concentrations were determined using HPV reference standards (1:40 and 1:30 starting dilutions with 2-fold titrations). A working concentration of 2.5 μg/mL for the Jackson and HP6043 conjugates was selected for further analysis so as to evaluate the accuracy by determining the ratio of the concentration results (Jackson/HP6043), with a standard point considered to be accurate when the value was within the range of 0.70 to 1.30.

### Assay validation.

The optimized HPV-9 cLIA and IgG-LIA were validated at Q^2^ Solutions Vaccines (formerly Focus Diagnostics, Inc.), San Juan Capistrano, CA, on behalf of Merck & Co. Inc., Rahway, NJ, USA. The validation studies evaluated various performance parameters, including the intra-assay precision (repeatability), intermediate precision, linearity, relative accuracy, and limits of quantitation (Table S1).

**(i) Linearity.** Assay linearity (i.e., the ability to elicit results that are proportional to the analyte concentration within a given range) was evaluated using 6 individual human serum samples from individuals who were vaccinated for all 9 HPV types. Each of the 6 samples was serially diluted 2-fold, with the number of dilutions being dependent on the antibody concentrations of the undiluted (i.e., neat) sample. The highest dilution targeted a concentration below the previously determined assay LLOQ, and the next highest dilution targeted the previously determined LLOQ. Each dilution was treated as an individual sample, and each neat/dilution sample was tested in single by 3 operators on 4 separate days (i.e., 12 total replicates per sample). Two lots of MS-VLP and 2 lots of either pooled 10× mAb-PE conjugate (for the HPV-9 cLIA validation) or goat anti-human IgG-PE conjugate (for the HPV-9 IgG-LIA validation) were evaluated. For each HPV type, a linear regression analysis was performed on the resulting series of measured concentrations for each sample. The acceptance criteria for each HPV type and sample required a slope of −1.25 to −0.80 and an R^2^ value of ≥0.95 for the HPV-9 cLIA and a slope of −1.20 to −0.90 and an R^2^ value of ≥0.95 for the IgG-LIA.

**(ii) Relative accuracy.** The relative accuracy, which refers to the ability of the assay to accurately quantitate fractions of a previously measured sample when those samples are spiked into a known negative sample or diluent at specific dilutions, was evaluated by using the results from the data set that was generated to assess the interassay linearity, as described above. Linear regression results were analyzed to estimate the bias per 10-fold dilution. The dilution bias was calculated by regressing the natural logarithm-transformed dilution corrected concentration at each dilution against the natural logarithm-transformed reciprocal dilution. The dilution bias per 10-fold dilution is (10*^b^*−1) ×100%, where *b* is the resulting slope estimate from the regression. For each HPV type, the mean estimate of the dilution bias is essentially obtained by taking the average of the slope estimates over the samples and runs and entering that average into the expression for dilution bias per 10-fold dilution. The assays were considered to be acceptably dilutable if the dilution bias per 10-fold dilution was ≤2.0-fold.

**(iii) Precision.** Precision refers to the degree of agreement among individual test results when a panel of samples spanning the quantifiable range is repeatedly assayed.

For both the HPV-9 cLIA and IgG-LIA, intra-assay precision (repeatability) was measured by comparing the results of samples tested under the same conditions (i.e., within the same assay run). Each of 5 samples from individuals who were vaccinated for all 9 HPV types, with antibody concentrations covering the range of the HPV-9 cLIA or IgG-LIA, were tested 10 times (20 wells; each sample in duplicate) in a single assay run by each of 2 operators, using the same MS-VLP and conjugate lots. The intra-assay %RSD was calculated for each sample for each HPV type and operator, and the median of these values was calculated to be the overall intra-assay %RSD for each HPV type. The validation acceptance criteria for cLIA and IgG-LIA intra-assay precision required %RSD values of ≤10% and ≤15%, respectively, for each HPV type.

Interassay precision (intermediate precision) involves measuring precision under conditions that vary during routine assay testing and performance (e.g., day, operator, and lots of MS-VLP and PE-labeled antibodies). To evaluate the interassay precision, a panel of 20 incurred human samples from individuals who were vaccinated for all 9 HPV types, with antibody concentrations across the range of the assay, was tested in single by 3 operators on 4 separate days (i.e., total of 12 replicates per sample). Two lots of MS-VLP and pooled mAb-PE conjugate (for the HPV-9 cLIA) or IgG-PE conjugate (for the HPV-9 IgG-LIA) were also incorporated. The interassay %RSD for each sample and the overall interassay %RSD (the median %RSD across the set of samples) were determined for each HPV type.

In addition to the 20 incurred samples, the interassay precision was assessed on the results from the serially diluted sample set that was referenced above as part of the linearity analysis. Each dilution of the 6 linearity samples was treated as an individual sample. The interassay %RSD for each sample and overall interassay %RSD (the median %RSD across the set of samples) were determined for each HPV type. For both the cLIA and IgG-LIA, the interassay precision validation acceptance criteria for each HPV type were a %RSD value of ≤30% RSD for ≥80% of the samples within the assays’ quantifiable range and an overall %RSD of ≤25%.

**(iv) Limits of quantitation.** The ULOQ and LLOQ were evaluated using the interassay precision incurred sample and linearity data sets. The test samples that were chosen for these data sets and the dilutions that were used to generate the linearity data set were chosen so as to result in concentrations across the assay range from the ULOQ to below the LLOQ. For each HPV type, the %RSD was calculated for all samples (neat and dilution) with antibody concentrations within the previously determined quantifiable range. The acceptance criteria required the %RSD to be ≤25% within the quantifiable range for each HPV type.

### Bridging to previous versions of the assays.

Bridging studies were performed to compare the results from the redeveloped assays with historical results that were generated with the previous assay versions (7, 9) in order to quantify the relationship between the 2 assays and to determine the concentrations in the redeveloped assays that correspond to the SSCOs for the previous assay versions. To this end, individual sera from participants from a 9vHPV vaccine clinical trial in young women aged 16 to 26 years (study V503-001; NCT00543543 [29, 31]) were tested using the redeveloped assays. The panel included randomly selected, deidentified, day 1 prevaccination, month 7 postvaccination, and month 36 long-term persistence serum samples from 100 participants as well as day 1 samples from an additional 50 participants. The day 1, month 36, and month 7 samples provided a range in antibody responses from negatives (i.e., below the SSCOs) to low positives (i.e., just above the SSCOs) to peak responses to the vaccine, respectively. The samples were tested using the updated assays at Q^2^ Solutions Vaccines, using a single lot of VLP-coupled beads, and the concentration results were compared with clinical results obtained using the previous versions of the assays. Summary statistics were calculated, and comparisons between the historical and new results were performed separately for each HPV type and by time of serum collection, post-vaccine dose. Comparisons between assay versions were performed on the natural logarithm-transformed concentrations.

For the purposes of estimating the concentration ratio between assay versions, concentrations reported as <LLOQ were excluded from the analysis. The functional relationship between the assay versions was estimated using the linear statistical relationship model of Tan and Iglewicz (32), which is a regression model that recognizes that measurement error is present in both assays that are being compared. Additionally, the relationship between assay measures was estimated by the Pearson correlation coefficient and Lin’s coefficients for accuracy and concordance (33).

### SSCO determination.

SSCOs were determined during the laboratory bridging study, using the panel of serum samples from the aforementioned study V503-001. For both assays, 2 × 2 cross-classification tables were created for each HPV type by serum collection time point, and they were constructed using the historic SSCO and alternative SSCO values for the updated assays. The negativity rate (i.e., 1 minus the positivity rate), agreement rate (i.e., the proportion of double-positive and double-negative samples, relative to the total number of samples), and McNemar’s exact *P*-value that resulted from the test for imbalance in discordant assignments were calculated for each cross-classification comparison.

For the HPV-9 cLIA, the SSCOs for the redeveloped assay were determined by balancing the discrepancy between the assay versions in the day 1 and month 36 positivity rates, with greater emphasis placed on the day 1 negativity rate. Mathematically, the SSCO was the value that minimized the serostatus cutoff function (*F_SSCO_*), which is defined as

$$F_{\mathrm{SSCO}}=0.6\times \left|\Delta_{D1}\right|+0.4\times \max\left(0,\Delta_{M36}\right),$$

where Δ*_D1_* and Δ*_M36_* denote the difference in the negativity rate between the cLIA versions (redeveloped version minus previous version) at day 1 and month 36, respectively. The expression gives 1.5 times the weight to minimizing the discrepancy on day 1, compared with month 36, and no weight is given to negative Δ*_M36_* values. To select among tied cutoff values, the values that provided for the best balance between the differences in the negativity rates at day 1 and month 36 were selected. The SSCOs were further refined, based on observations using clinical study data that were generated with the revised assays during routine assay testing and following the subsequent re-evaluation of the bridging study so as to better align the seropositivity rates between the assay versions.

For the HPV-9 IgG-LIA, the following criteria were used for the selection of the SSCOs for each HPV type: (i) it must exceed the LLOQ by ≥1 mMU/mL and (ii) the negativity rate for day 1 is within 1.0 percentage point of the negativity rate for the day 1 samples in the prior version of the assay.

### HPV-16 and HPV-18 reference standard evaluation.

International reference standards containing HPV-16-specific and HPV-18-specific antibodies were obtained from the National Institute for Biological Standards and Control (NIBSC). On each of 3 days, international reference standards were reconstituted, per vendor instructions, and diluted to the minimum required dilution (MRD) of the respective assay (1:10 cLIA and 1:100 IgG-LIA). The international standards were then serially diluted 1:1.5 across 6 wells of a 96-well plate. On the same plates, MSD’s internal reference standards were diluted to MRD and were further serially diluted, per the respective test methods, across the 12 plate columns (1:1.7 cLIA and 1:2.1 IgG-LIA). Each reference standard was prepared twice, in duplicate, per plate, with two plates being run for each assay on each day. Following the reference standard preparation, the remainder of the assays were executed per the current test methods.
