# Supplementary material for: Development and Validation of Two Optimized Multiplexed Serologic Assays for the 9-Valent Human Papillomavirus Vaccine Types
Source: mSphere. 2023 Mar 16;8(2):e00962-21. doi: 10.1128/msphere.00962-21 (PMC10117101; doi:10.1128/msphere.00962-21)
Supplement: TABLE S1 [file msphere.00962-21-s0001.docx]

| **Parameter** | **Description** | **^a^ Acceptance criteria** | **Results** |
| --- | --- | --- | --- |
| ***HPV-9 cLIA*** |  |  |  |
| **Reference standards and control samples** | Standard curve and 4 controls were evaluated to confirm plate acceptance criteria for bead replicate variability, RMSE, and control range concentrations. | Standards and controls must meet prespecified validity criteria (RMSE 3σ upper limits for reference standards and acceptable ranges for the controls), as determined during assay qualification.  Control sample variability should be consistent with that of test samples. | Standard curve and 4 controls included on all plates tested in the validation study.  Standard curves were evaluated for both RMSE and the number of valid standard curve points. All control results were evaluated against the 2σ control limits established during assay qualification. |
| **Intra-assay precision (repeatability)** | Each of 5 samples from individuals vaccinated for all 9 HPV types (positive for all 9 HPV types) with antibody concentrations covering the range of the assay were tested 10 times in a single assay run by each of 2 operators. | %RSD must be ≤10% for each HPV type. | Intra-assay precision (%RSD) was <10% for 70 of the 88 evaluable combinations of sample, operator, and HPV type.  The overall (combined over all samples within an HPV type) intra-assay precision/repeatability was <10% for each HPV type. |
| **Inter-assay precision (intermediate precision)** | A minimum of 6 dilutions of 6 individual high-concentration samples from individuals vaccinated for all 9 HPV types (positive for all 9 HPV types) were tested in singleton by 3 operators on each of 4 separate days for a total of 12 replicates for each neat sample and each dilution sample. The number of dilutions was dependent on the antibody concentrations of the undiluted (neat) sample. The highest dilution targeted a concentration below the LLOQ of the assay and the next-to-highest dilution targeted the LLOQ of the assay.  Two lots of MS-VLP and 2 lots of pooled mAb-PE conjugate were also incorporated into this study. | Assay precision ≤30% RSD for ≥80% of samples with median antibody concentrations within the assay’s quantifiable range.  Overall precision (%RSD) ≤25%.  Ratio of antibody concentrations determined between MS-VLP or mAb-PE lots of <1.3-fold on average for each pairwise comparison. | 307 (97.8%) of 314 samples having an antibody concentration within the quantifiable range had %RSD ≤30%.  For each HPV type, the overall precision (%RSD) was <9%.  A variance component analysis was performed to quantify the different sources of assay variability. For each HPV type, the %RSD was under 6% for each of the ruggedness factors (Operator, MS-VLP lot, mAb-PE lot) and the overall estimate of intermediate precision was <14%.  The ratio of cLIA concentrations between 2 lots of mAb-PE was within ±1.04-fold on average for each HPV type.  The ratio of cLIA concentrations between 2 MS-VLP lots was within ±1.04-fold on average for each HPV type. |
| **Linearity** |  | Slope within range of ‑0.80 to -1.25 and R^2^ value ≥0.95 for all HPV types for each sample. | 641 of 648 individual test sample dilution curves had a slope that was within the range of -0.80 and -1.25, and all 648 individual curves had an R^2^ value >0.97. When averaged over the runs, the slope was within the range of -0.80 and -1.25 for each sample and each HPV type. |
| **Relative accuracy** |  | Considered acceptably dilutable if the dilution-bias per 10-fold dilution is ≤2.0-fold. | The estimated dilution bias was ≤2.0-fold per 10-fold dilution for 641 of 648 individual estimates of dilution bias. For each HPV type, the overall estimate of dilution bias (combined over all samples and runs within an HPV type) was within 1.25-fold per 10-fold dilution. |
| **LLOQ/ULOQ (dilution and incurred samples)** |  | %RSD ≤25% within the quantifiable range of the assay for each HPV type:  HPV6: 20–1005 mMU/ml  HPV11: 16–664 mMU/ml  HPV16: 20–3581 mMU/ml  HPV18: 24–956 mMU/ml  HPV31: 10–875 mMU/ml  HPV33: 8–474 mMU/ml  HPV45: 8–351 mMU/ml  HPV52: 8–432 mMU/ml  HPV58: 8–546 mMU/ml | Of 314 dilution samples having a concentration within the quantifiable range, 299 (95.2%) had %RSD ≤25%.  The overall (over all dilution samples within an HPV type) precision (%RSD) for the dilution samples was <9% for each HPV type.  All 176 incurred samples having a concentration within the quantifiable range had %RSD ≤20%. The overall (over all incurred samples within an HPV type) precision (%RSD) for the incurred samples was <11% for each HPV type. |
| **Inter-assay precision (incurred samples)** | Twenty human samples from individuals vaccinated for all 9 HPV types (positive for all 9 HPV types) with antibody concentrations across the range of the assay were tested in singleton by 3 operators on each of 4 separate days for a total of 12 replicates for each incurred sample.  Two lots of MS-VLP and 2 lots of pooled mAb-PE conjugate were incorporated into this study. | Assay precision ≤30% RSD for ≥80% of samples.  Overall precision (%RSD) ≤25%.  Ratio of antibody concentrations between lots of MS-VLP or mAb-PE <1.3-fold on average for each pairwise comparison. | For each HPV type, every sample (100%) having an antibody concentration within the quantifiable range had %RSD <20%.  For each HPV type, the overall precision (%RSD) was <11%.  Additionally, a variance component analysis was performed to quantify the different sources of assay variability. For each HPV type, the %RSD was negligible for each of the ruggedness factors (Operator, MS-VLP lot, mAb-PE lot) and the overall estimate of intermediate precision was <11%.  The ratio of cLIA concentrations between 2 mAb-PE lots was within ±1.03-fold on average for each HPV type. The ratio of cLIA concentrations between 2 MS-VLP lots was within ±1.04-fold on average for each HPV type. |

| ***HPV-9 IgG-LIA*** |  |  |  |
| --- | --- | --- | --- |
| **Reference standards and control samples** | Standard curve and 4 controls were evaluated to confirm plate acceptance criteria for bead replicate variability, RMSE, and control range concentrations. | Standards and controls must meet prespecified validity criteria.  Control sample variability should be consistent with that of test samples. | Of 41 assay plates generated as part of the validation study, only 1 plate failed because of having an insufficient number of valid standard curve points and ≥2 failed controls. All other assay plates met the reference standard curve and control acceptance criteria. |
| **Intra-assay precision (repeatability)** | Each of 5 samples from individuals vaccinated for all 9 HPV types (positive for all 9 HPV types) with antibody concentrations covering the range of the assay were tested 10 times in a single assay run by each of two operators. | %RSD must be ≤15% for each HPV type. | Intra-assay precision (%RSDs) was <7% for each individual sample.  The overall (combined over all samples within an HPV type) intra-assay %RSD was <4% for each HPV type. |
| **Inter-assay precision (intermediate precision)** | A minimum of 6 dilutions of 6 individual high-concentration samples from individuals vaccinated for all 9 HPV types (positive for all 9 HPV types) were tested in singleton by 3 operators on each of 4 separate days for a total of 12 replicates for each neat sample and each dilution sample. The number of dilutions was dependent on the antibody concentrations of the undiluted (neat) sample. The highest dilution targeted a concentration below the LLOQ of the assay and the next-to-highest dilution targeted the LLOQ of the assay.  Two lots of MS-VLP and 2 lots of conjugate were incorporated into this study. | Assay precision ≤30% RSD for ≥80% of samples with median antibody concentrations within the assay’s quantifiable range.  Overall precision (%RSD) ≤25%.  Ratio of antibody concentrations determined between MS-VLP or conjugate lots of <1.3-fold on average for each pairwise comparison. | For each HPV type, every sample having an antibody concentration within the quantifiable range had %RSD <20%.  For each HPV type, the overall precision (%RSD) was <8%.  A variance component analysis was performed to quantify the different sources of assay variability. For each HPV type, the %RSD was under 3% for each of the ruggedness factors (Operator, MS-VLP lot, IgG-PE lot) and the overall estimate of intermediate precision was <9%.  The ratio of IgG concentrations between 2 lots of IgG-PE was within ±1.06-fold on average for each HPV type.  The ratio of IgG concentrations between 2 MS-VLP lots was within ±1.02-fold on average for each HPV type. |
| **Linearity** |  | Slope within range of ‑0.90 to ‑1.20 and R^2^ value ≥0.95 for all HPV types for each sample. | Each of 639 individual test sample dilution curves had a slope that was within the range of -1.01 and -1.15, and an R^2^ value >0.99. When averaged over the runs, the slope was within the range of -0.90 and -1.20 for each sample and each HPV type. |
| **Relative accuracy** |  | Considered acceptably dilutable if the dilution-bias per 10-fold dilution is ≤2.0-fold. | The estimated dilution bias was ≤2.0-fold per 10-fold dilution for all 639 individual estimates of dilution bias. For each HPV type, the overall estimate of dilution bias (combined over all samples and runs within an HPV type) was within 1.25-fold per 10-fold dilution. |
| **LLOQ/ULOQ (dilution and incurred samples)** |  | %RSD ≤25% within the quantifiable range of the assay for each HPV type:  HPV6: 2–500 mMU/ml  HPV11: 2–300 mMU/ml  HPV16: 4–1700 mMU/ml  HPV18: 3–540 mMU/ml  HPV31: 2–400 mMU/ml  HPV33: 2–270 mMU/ml  HPV45: 1–200 mMU/ml  HPV52: 1–200 mMU/ml  HPV58: 2–250 mMU/ml | For each HPV type, the %RSD was <20% for each sample having an antibody concentration within the quantifiable range of the assay.  The overall (over all dilution samples within an HPV type) precision (%RSD) for the dilution samples was <8% for each HPV type.  All incurred samples having a concentration within the quantifiable range had %RSD ≤12%. The overall (over all incurred samples within an HPV type) precision (%RSD) for the incurred samples was <8% for each HPV type. |
| **Inter-assay precision (incurred samples)** | Twenty human samples from individuals vaccinated for all 9 HPV types (positive for all 9 HPV types) with antibody concentrations across the range of the assay were tested in singleton by 3 operators on each of 4 separate days for a total of 12 replicates for each incurred sample.  Two lots of MS-VLP and 2 lots of pooled IgG-PE conjugate were incorporated into this study. | Assay precision ≤30% RSD for ≥80% of samples.  Overall precision (%RSD) ≤25%.  Ratio of antibody concentrations between lots of MS-VLP or conjugate <1.3-fold on average for each pairwise comparison. | For each HPV type, every sample (100%) having an antibody concentration within the quantifiable range had %RSD <12%.  For each HPV type, overall precision (%RSD) was <8%.  Additionally, a variance component analysis was performed to quantify the different sources of assay variability. For each HPV type, the %RSD was negligible for each of the ruggedness factors (Operator, MS-VLP lot, IgG-PE lot) and the overall estimate of intermediate precision was <7%.  The ratio of IgG concentrations between two IgG-PE lots was within ±1.03-fold on average for each HPV type. The ratio of IgG concentrations between 2 MS-VLP lots was within ±1.03-fold on average for each HPV type. |
